# The Role of Inspiratory Muscle Training in Sickle Cell Anemia Related Pulmonary Damage due to Recurrent Acute Chest Syndrome Attacks

**DOI:** 10.1155/2015/780159

**Published:** 2015-04-28

**Authors:** Burcu Camcıoğlu, Meral Boşnak-Güçlü, Müşerrefe Nur Karadallı, Şahika Zeynep Akı, Gülsan Türköz-Sucak

**Affiliations:** ^1^Department of Physiotherapy and Rehabilitation, Faculty of Health Sciences, Gazi University, 06500 Ankara, Turkey; ^2^Department of Hematology, Faculty of Medicine, Gazi University, 06500 Ankara, Turkey

## Abstract

*Background*. The sickling of red blood cells causes a constellation of musculoskeletal, cardiovascular, and pulmonary manifestations. A 32-year-old gentleman with sickle cell anemia (SCA) had been suffering from recurrent acute chest syndrome (ACS). *Aim*. To examine the effects of inspiratory muscle training (IMT) on pulmonary functions, respiratory and peripheral muscle strength, functional exercise capacity, and quality of life in this patient with SCA. *Methods*. Functional exercise capacity was evaluated using six-minute walk test, respiratory muscle strength using mouth pressure device, hand grip strength using hand-held dynamometer, pain using Visual Analogue Scale, fatigue using Fatigue Severity Scale, dyspnea using Modified Medical Research Council Scale, and health related quality of life using European Organization for Research and Treatment of Cancer QOL measurement. *Results*. A significant improvement has been demonstrated in respiratory muscle strength, functional exercise capacity, pain, fatigue, dyspnea, and quality of life. There was no admission to emergency department due to acute chest syndrome in the following 12 months after commencing regular erythrocytapheresis. *Conclusion*. This is the first report demonstrating the beneficial effects of inspiratory muscle training on functional exercise capacity, respiratory muscle strength, pain, fatigue, dyspnea, and quality of life in a patient with recurrent ACS.

## 1. Introduction

Sickle cell anemia (SCA) is a hereditary form of hemolytic anemia characterized by production of abnormal hemoglobin (HbS). The sickling of red cells causes a constellation of musculoskeletal, cardiovascular, and pulmonary manifestations including palpitations, dyspnea, fatigue, and pain; these symptoms worsen during physical effort [1].

Multiple complications such as vasoocclusive painful crisis, acute chest syndrome, pulmonary hypertension, and stroke occur in SCA [2]. Acute chest syndrome (ACS) is one of the most important SCA related events which is a form of vasoocclusive crisis characterized by pulmonary manifestations such as fever, cough, tachypnea, chest pain, hypoxia, and infiltrations on chest X-rays. Two or more attacks per year are designated as recurrent acute chest syndrome [3].

Exercise capacity decreases in patients with SCA, as a result of reduced oxygen carrying capacity related to high HbS levels, functional and structural cardiac adaptations resulting from chronic anemia, pulmonary dysfunction caused by repeated episodes of acute chest syndrome, and peripheral vascular impairment due to microvascular occlusion [4].

There is no study investigating the effects of inspiratory muscle training in patients with SCA and lung injury to our knowledge. The aim of the present study was to investigate the effect of inspiratory muscle training (IMT) on respiratory muscle strength, functional exercise capacity, dyspnea, pain and fatigue perception, and quality of life in this patient with SCA with recurrent ACS.

## 2. Case Presentation

A 32-year-old male patient, who was diagnosed with sickle cell anemia at the age of 2, has had recurrent acute chest attacks in the following years. He remained on hydroxyurea (500 mg, 2 doses/day) between the ages 17 and 24 until he refused to take the drug anymore. He had no acute chest attack under hydroxyurea treatment. Three years after quitting the medical treatment he had his first acute chest attack in 2007 after quitting hydroxyurea. He suffered from acute chest syndrome for the following two weeks. He had erythrocytapheresis (ECP) implemented and acute chest attack was cured after the process. He was started on regular ECP every 8–12 weeks in 2008 to prevent the attacks. He had a history of repeating admissions to the emergency department due to painful vasoocclusive crises and acute chest syndrome. From 2008 until starting the IMT he was admitted to emergency department with vasoocclusive crisis for 7 times and with ACS for 4 times. Crisis occurred with a median of 2 attacks per year (range 1 to 3). Under hydroxyurea treatment he had a Hb F level of median 2,1% (range 0,1% to 6,9%). He was complaining of exertional dyspnea which withheld him from promoting moderate physical activity. Measurements were performed before and after all ECP sessions. Inspiratory muscle training was started after the first ECP session and continued for 12 weeks.

## 3. Methods

### 3.1. Erythrocytapheresis

Erythrocytapheresis was performed by an automated red cell exchange program using COBE Spectra apheresis system. Whole blood: ACD ratio was 13 : 1 and one volume of the patient's red cells was exchanged via peripheral venous cannula at 8–12-week intervals. Prior to initiation of each ECP, red cell phenol typing, group, and antibody screening were performed. The hematocrit in the red blood cell line was correlated with the red cell concentrates transfused. The net red blood cell mass/kg was calculated for each procedure based on the measured hematocrit of the patient's weight, height, and gender.

### 3.2. Pulmonary Function Tests

Spirometric measurement was performed using a portable spirometry (Vmax 220 SensorMedics Corporation, Yorba Linda, CA) according to the guidelines of the American Thoracic Society [5].

### 3.3. Respiratory Muscle Strength

Maximal inspiratory pressure (MIP) and maximal expiratory pressure (MEP) were assessed using an electronic pressure transducer (MicroRPM, Micromedical, Kent, UK) according to ATS/ERS (American Thoracic Society/European Respiratory Society) [6] The minimal clinically important significance (MCID) for inspiratory muscle strength was 11 cmH_2_O [7].

### 3.4. Hand Grip Strength

Hand grip strength was measured using hand-held dynamometer (JTECH PowerTrack Commander II, USA) [8].

### 3.5. Functional Exercise Capacity

Six-minute walk test (6MWT) was implemented in a 30-m corridor according to the guidelines of the ATS/ERS [9]. Heart rate was monitored with a heart rate monitor (Polar FT1 Electro Oy, Finland) during the test. Peak heart rate values achieved during the tests were recorded. Modified Borg Dyspnea Scale was used before and after the 6MWT. The MCID for 6MWT was 25 m [10].

### 3.6. Fatigue

Fatigue was evaluated using Turkish version of Fatigue Severity Scale (FSS) [11].

### 3.7. Pain

Pain was measured using Visual Analog Scale (VAS) [12].

### 3.8. Dyspnea

Modified Medical Research Council (MMRC) dyspnea scale was used to evaluate dyspnea severity during activity. Levels of dyspnea are graded from 0 (absence of dyspnea during strenuous exercise) to 4 (dyspnea during daily activities) [13].

### 3.9. Quality of Life

Health related quality of life (HRQOL) was assessed using Turkish version of the European Organization for Research and Treatment of Cancer QOL measure (EORTC QLQ-C30). All functional scales and individual item scores are transformed to a 0–100 scale with higher values indicating a higher functioning in functional scales and an increased presence of symptoms in symptom scales [14]. The MCID was 16.8 on social functioning, 4.6 on global health status, and (−) 11.4 on fatigue [15].

### 3.10. Inspiratory Muscle Training Protocol

Patient received IMT at 30% of MIP using PowerBreathe device (wellness/green) (IMT Technologies, Warwickshire). MIP was measured at supervised sessions and training loads were adjusted weekly to maintain 30% of the MIP. Patient was trained for 30 min per day, 7 days per week, for 12 weeks. Patient's heart rate, blood pressure, and breathing frequency were monitored during the IMT sessions once a day each week. Patient was instructed to maintain diaphragmatic breathing and try to maintain 10–15 breaths and rest 5–10 seconds between breaths.

## 4. Results

Patient is a thirty-three-year-old overweight gentleman, an ex smoker, who gave up smoking 3 months ago. Baseline pulmonary function testing revealed both obstructive and restrictive pulmonary function abnormalities. Obstruction in small airways and decrease in pulmonary diffusion capacity were also demonstrated (Table 1).

Hb S level was reduced to below the target level of 30% in each ECP session. Hepatitis B, C and HIV test and alloantibody panel was repeated regularly and no infectious and immunologic complications occurred so far. His ferritin level which was 1230 ng/mL before the first ECP session was 495 ng/mL a year after commencing ECP.

**Figure 1:**
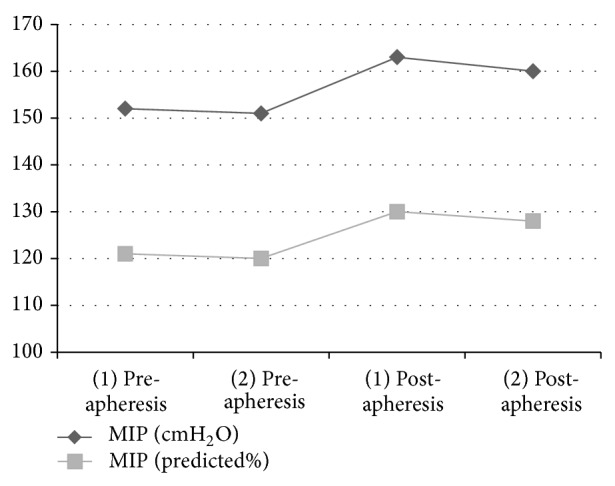
Pre- and postapheresis improvements of inspiratory muscle strength.

The inspiratory muscle training was started after the 1st ECP session and continued for 12 weeks. MIP (11 cmH_2_O, 152 cmH_2_O to 163 cmH_2_O), MIP% (9%, 121% to 130%) (Figure 1), MEP (12 cmH_2_O, 178 cmH_2_O to 190 cmH_2_O), and MEP% (6%, 87% to 93%) improved when the 1st and 2nd ECP session respiratory muscle strength values were compared (Table 2).

Although his baseline MIP and MEP values were not below 80 cmH_2_O (indicating a respiratory muscle weakness), inspiratory muscle strength improvement (11 cmH_2_O, 152 cmH_2_O to 163 cmH_2_O) was clinically significant after IMT.

Hand grip muscle strength also increased (34.7 to 35.8 kgf) after IMT though without clinical significance (Table 2).

**Figure 2:**
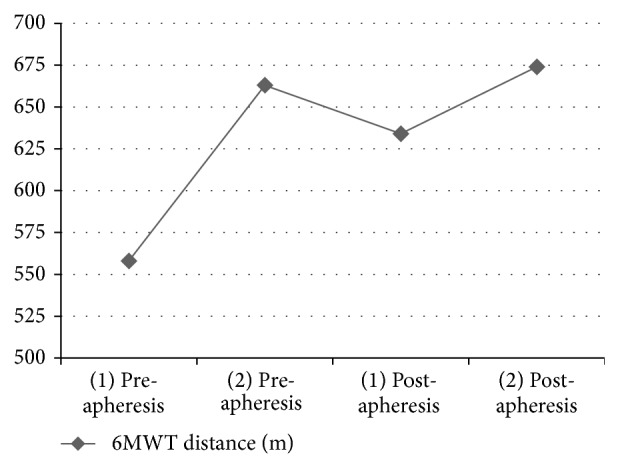
Pre- and postapheresis improvements of six-minute walk test.

The distance covered during 6MWT (105 m, 558 m to 663 m) and 6MWT% (13%, 72% to 85%) improved after the first ECP. The 6MWT (30 m, 634 m to 674 m) and 6MWT% (5%, 82% to 87%) further improved after the second ECP. ECP significantly improved the patient's 6MWT distance and the distance covered by the patient decreased gradually until the next session. The distance covered during 6MWT (76 m, 558 m to 634 m) and 6MWT% (10%, 72% to 82%) (Figure 2) improved and was over MCID of 6MWT when the 1st and 2nd ECP values were compared. This improvement, which is clinically important, was attributed to IMT, as the 6MWT distance which the patient covered before 1st and 2nd ECP would be expected to be the same in normal circumstances (Table 3).

Resting heart rate decreased and heart rate response during the exercise improved after IMT. The patient reached maximal heart rate during all 6MWTs. After IMT heart rate, during submaximal exercise test tended to decrease as the patient walked more distance (Table 3).

There was no difference in dyspnea perception after the 1st ECP; however, there was a decrease in dyspnea perception (2 to 0) between 1st and 2nd ECP session and it was over MCID of MMRC when the 1st and 2nd ECP values were compared. Fatigue and pain scores decreased. There was no pain perception during resting and the improvement of pain was over MCID after IMT. The vasoocclusive crises did not repeat after starting ECP (Table 4).

Global health status, functional scales, and symptom scales improved and fatigue reduced after IMT. Improvements on global health status (13.7, 50 to 66.7) after the 2nd ECP session were clinically significant and fatigue (11.2, 55.6 to 44.4) was close to the minimal clinical significance (Table 5).

## 5. Discussion

The present study demonstrates the clinical improvement in functional capacity (6MWT: 76 m), respiratory muscle strength (MIP: 11 cmH_2_O, MEP: 35 cmH_2_O), health related quality of life (global health status: 33.4), dyspnea (MMRC: 2), fatigue (FSS: 11), and pain (VAS: 5.5 cm) perception after IMT. No adverse effects or a painful crisis was seen during IMT.

Improvement in objective and subjective outcomes, such as respiratory muscle strength, functional exercise capacity, quality of life, pain, dyspnea, and fatigue, has been documented.

Abnormal pulmonary functions characterized by airway obstruction, restrictive lung disease, abnormal diffusing capacity, and hypoxemia reflect the lung damage in SCA patients and occur in up to 90% of patients [16]. The presented patient had obstructive and restrictive pulmonary function patterns and also obstruction in small airways and decreased pulmonary diffusion capacity, similar to the pulmonary function abnormality pattern.

In a similar previous report a 32-year-old patient who was frequently hospitalized for infections and had high levels of pain, fatigue, exertional dyspnea, and leg ulcers had an inspiratory muscle strength below 80 cmH_2_O (76.7 cmH_2_O). This patient was reported to experience decrease in pain, increase in respiratory muscle strength (76.7 cmH_2_O to 79.2 cmH_2_O), and improvement in quality of life after aquatic rehabilitation interventions [1]. Although our patient had no respiratory muscle weakness, patient's respiratory muscle strength improved and also he had objective and subjective clinical manifestations which improved significantly after IMT. Nevertheless inspiratory muscle training leads to improvement in not only respiratory muscle strength but also exercise capacity, quality of life, peripheral muscle strength, peak VO_2_, and oxygen uptake efficiency in patients with chronic obstructive pulmonary disease and heart failure [17, 18].

Although IMT was reported to improve the respiratory muscle metaboreflex by eliciting adequate blood flow to the peripheral muscles [19], the peripheral muscle strength determined with measuring hand grip muscle strength which was decreased in our patient did not show any improvement after IMT.

The functional exercise capacity of our patient was impaired (558 m, 72% < 80% predicted). Patients with SCA are well known to have decreased exercise capacity [19] and reached lower power output and VO_2_ at ventilatory threshold during submaximal exercise testing compared with healthy controls [20]. Chronic anemia, acute chest syndrome, restrictive lung disease, and pulmonary vascular disease were claimed to contribute to the limited exercise capacity in SCA patients and improve significantly with exchange transfusion [5, 19, 20].

The predicted 6MWT% was 72% in our patient before training and he had a decreased exercise capacity. Our previous experience in patients with heart failure [21] and previous reports in chronic pulmonary disease [22] inspired us to investigate whether IMT would improve the exercise capacity and objective symptoms of the current patient. His 6MWT distance showed a significant improvement and reached 674 m after IMT. There was also a clinically significant improvement in the 6MWT distance (76 m, 558 m to 634 m) and 6MWT% (10%, 72% to 82%) which was over MCID of 6MWT. We also demonstrated clinically significant improvement (76 m MICD: 25 m) in exercise capacity between two apheresis sessions which we attribute to IMT.

SCA patients have a higher heart rate during resting and exercise which might be interpreted as a physiological adaptation to compensate for the decreased blood oxygen transport capacity related to anemia and lower oxygen saturation [23]. We believe IMT at least contributed to the improvement in heart rate during resting and after exercise testing.

Dyspnea and fatigue are frequent in patients with SCA and various pathophysiologic mechanisms might be involved besides anemia such as inflammation, pain, sleep quality, anxiety, depression, and stress. Our patient experienced a significant improvement in fatigue and pain perception and his dyspnea had also improved. He declares climbing, walking, running, and doing the daily activities were much more comfortable. IMT seems to be a promising and effective method to decrease fatigue, pain, and dyspnea.

In accordance with the literature our patient showed a poor HRQOL and lower levels of vitality/energy than the general population and other chronic illnesses [24]. We found similar results on all domains (except mental health) especially vitality domain. He had worse quality of life before the training, which improved significantly after ECP and IMT. Similar improvements have been shown in patients with COPD and heart failure [17, 21].

Previous studies have demonstrated concrete effectiveness of regular ECP on SCA related acute events including acute chest syndrome [25]. Our patient did not have any SCA related acute events after commencement of ECP, which he tolerated very well. He had no infectious, immunologic complications and had a negligible iron overload with ferritin levels around 500 ng/mL which did not require iron chelation. Regular ECP induces increase in oxygenation and decreases HbS [26]. The improvement of blood rheology with ECP resulted in decrease in pain and fatigue and improvement in functional exercise capacity, dyspnea, and quality of life. We started ECP for preventing further lung injury; however, he also had preexisting functional impairment. A well established pulmonary rehabilitation program which included inspiratory muscle training has been very beneficial, appropriate, and simple as an auxiliary treatment modality in parallel with ECP in improving this functional impairment. There is a need to determine an effective exercise type and rehabilitation program which would have beneficial effects on functional exercise capacity and respiratory muscle strength that SCA patients would benefit from, without any risk of evoking vasoocclusive crisis. Interventions should target decreasing pain, fatigue, and dyspnea and improving exercise capacity, respiratory and peripheral muscle strength, and health related quality of life. The present study showed that inspiratory muscle training is an important strategy for the improvement of HRQOL, exercise capacity, respiratory muscle strength, pain, dyspnea, and fatigue perception and no adverse effects occurred during treatment. We hope our report will inspire further prospective studies with larger sample size which investigates the role of inspiratory muscle training in SCA patients.

## Figures and Tables

**Table 1:** Demographic characteristics.

Characteristics	Variable
Age (years)	32
Weight (kg)	92
Height (cm)	183
BMI (kg/m^2^)	27.47
Smoking (packxyears)	4.6
Disease duration (years)	30
FVC (%)	56
FEV_1_ (%)	53
FEV_1_/FVC	79
PEF (%)	64
FEF_25–75%_ (%)	45
DLCO (%)	62

FVC: forced vital capacity; FEV_1_: forced expiratory volume in one second; PEF: peak expiratory flow; FEF_25–75%_; DLCO: carbon monoxide diffusing capacity.

**Table 2:** Pre- and postapheresis results of respiratory and peripheral muscle strength.

Characteristics	(1)		(2)	
	Preapheresis	Postapheresis	Preapheresis	Postapheresis
MIP (cmH_2_O)	152	151	163	160
MIP (predicted%)	121	120	130	128
MEP (cmH_2_O)	178	181	190	213
MEP (predicted%)	87	89	93	104
Hand grip (kgf)	34.7	35.6	33.5	35.8

MIP: maximal inspiratory pressure; MEP: maximal expiratory pressure.

**Table 3:** Pre- and postapheresis values of 6-minute walk test distance and hemodynamic responses.

Characteristics	(1)		(2)	
	Preapheresis	Postapheresis	Preapheresis	Postapheresis
6MWT (m)	558	663	634	674
6MWT (predicted%)	72	85	82	87
Heart rate (beats/min, before)	112	94	91	78
Heart rate (beats/min, after)	170	186	192	160
ΔHeart rate (beats/min)	58	92	101	82

6MWT: six-minute walk test.

**Table 4:** Pre- and postapheresis values of dyspnea, fatigue, and numbers of vasoocclusive crises and emergency department admission.

Characteristics	(1)		(2)	
	Preapheresis	Postapheresis	Preapheresis	Postapheresis
MMRC (0–4)	2	2	2	0
Fatigue Severity Scale (0–63)	60	53	55	49
Pain, mm (0–100)	55	3	42	0
Vasoocclusive crises (*n*)	2	0	0	0
Emergency service admission (*n*)	2	0	0	0

MMRC: Modified Medical Research Council Scale.

**Table 5:** Pre- and postapheresis values of EORTC QLQ-C30 health related quality of life survey.

EORTC QLQ-C30	(1)		(2)	
	Preapheresis	Postapheresis	Preapheresis	Postapheresis
Global health status (0–100)	33.3	50	33.3	66.7
Functional scales (0–100)	68.9	66.7	68.9	71.1
Symptom scales (0–100)	28.2	30.7	30.7	28.2
Social functioning (0–100)	83.3	100	83.3	83.3
Fatigue (0–100)	44.4	55.6	33.3	44.4

EORTC QLQ-C30: European Organization for Research and Treatment of Cancer QOL measure.
